# Referral, treatment patterns and change in quality of life in the first 12 months in children and young people with juvenile idiopathic arthritis: an analysis of the association with ethnicity and socioeconomic position using data from a cohort study

**DOI:** 10.1093/rheumatology/keag332

**Published:** 2026-06-25

**Authors:** Richard P Beesley, Alice Chieng, Coziana Ciurtin, Gavin Cleary, Flora McErlane, Stephanie J W Shoop-Worrall, Lucy R Wedderburn, Kimme L Hyrich, Jenny H Humphreys, Lianne Kearsley-Fleet

**Affiliations:** Versus Arthritis Centre for Epidemiology, Centre for Musculoskeletal Research, Division of Musculoskeletal and Dermatological Sciences, The University of Manchester, Manchester Academic Health Science Centre, Manchester, UK; Department of Rheumatology, Manchester Children’s Hospital, University of Manchester, Manchester, UK; Centre for Adolescent Rheumatology, Department of Ageing, Rheumatology and Regenerative Medicine, Division of Medicine, University College London, London, UK; Department of Rheumatology, Alder Hey Children’s Hospital, Liverpool, UK; Newcastle University Population Health Sciences Institute, Great North Children’s Hospital, Newcastle upon Tyne, UK; Versus Arthritis Centre for Epidemiology, Centre for Musculoskeletal Research, Division of Musculoskeletal and Dermatological Sciences, The University of Manchester, Manchester Academic Health Science Centre, Manchester, UK; Infection, Immunity and Inflammation Research and Teaching Department, University College London Great Ormond Street Institute of Child Health, London, UK; National Institute of Health Research Biomedical Research Centre, Great Ormond Street Hospital, London, UK; Centre for Adolescent Rheumatology, University College London, London, UK; Versus Arthritis Centre for Epidemiology, Centre for Musculoskeletal Research, Division of Musculoskeletal and Dermatological Sciences, The University of Manchester, Manchester Academic Health Science Centre, Manchester, UK; National Institute of Health Research Manchester Biomedical Research Centre, Manchester University NHS Foundation Trust, Manchester, UK; Versus Arthritis Centre for Epidemiology, Centre for Musculoskeletal Research, Division of Musculoskeletal and Dermatological Sciences, The University of Manchester, Manchester Academic Health Science Centre, Manchester, UK; National Institute of Health Research Manchester Biomedical Research Centre, Manchester University NHS Foundation Trust, Manchester, UK; Versus Arthritis Centre for Epidemiology, Centre for Musculoskeletal Research, Division of Musculoskeletal and Dermatological Sciences, The University of Manchester, Manchester Academic Health Science Centre, Manchester, UK

**Keywords:** ethnicity, socioeconomic position, juvenile idiopathic arthritis, disease activity, psychosocial, referral patterns, time to treatment

## Abstract

**Objectives:**

Delays in JIA diagnosis may impact long-term outcomes and health-related quality of life (HRQoL). This study investigated referral pathways, initial treatment patterns and changes in HRQoL among UK children and young people with JIA stratified by ethnic group and socioeconomic position.

**Methods:**

Treatment-naïve children and young people diagnosed with JIA between 2001 and 2019 were recruited into the Childhood Arthritis Prospective Study (CAPS). Outcomes included duration to first appointment, and change in HRQoL and disease activity following initial presentation. Associations between duration, presentation and referral source with ethnicity and socioeconomic position, were assessed using adjusted Cox proportional hazards models.

**Results:**

Of 1275 patients, 65% were female, 91% White ethnicity (5% Asian, 1% Black, 3% Mixed) and 27% in the most deprived socioeconomic group. Median age was 8 years. Median time to first appointment was 18 weeks, varying slightly by ethnic group but not socioeconomic position. Referral source and initial treatment were similar across groups. While disease activity and HRQoL improved over the first 12 months following first appointment in paediatric rheumatology for individuals with JIA, the overall HRQoL measures remained below population norms for people without JIA, with limited improvement beyond 1 year. Ethnicity and socioeconomic position were not associated with time to appointment, initial treatment or changes in disease activity or HRQoL.

**Conclusion:**

In this national inception cohort, ethnicity and socioeconomic position do not appear to be associated with access to specialist care, initial treatment or early clinical outcomes. Persistent low HRQoL despite reduced disease activity highlights the need to address physical and psychosocial wellbeing beyond disease control.

## Graphical abstract

**Graphical Abstract:**
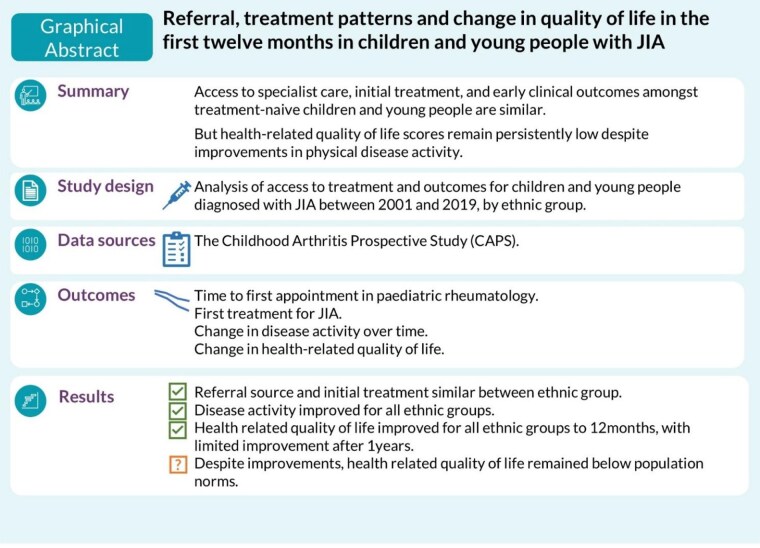

Rheumatology key messages

- Ethnicity and socioeconomic position do not appear to influence access to specialist care, initial treatment or early clinical outcomes amongst treatment-naïve children and young people diagnosed with JIA.
- Health-related quality of life scores remain persistently low despite improvements in physical disease activity.
- Further research to understand this phenomenon is warranted, to support targeted interventions to improve overall quality of life for these patients.

## Introduction

JIA is a collection of related autoimmune conditions characterized by chronic joint inflammation of unknown cause with onset prior to 16 years of age [1]. Overall prevalence of JIA is around 1 in 1600 children and young people in the UK [2].

JIA can lead to joint damage, chronic pain and potentially permanent disability, impacting both physical and mental health, as well as social and economic prospects [3, 4]. However, advances in treatment options have generally led to improved patient outcomes overall.

Previous research suggests there may be differences in the incidence and prevalence of JIA between different ethnic groups in the UK [5]. However, less is known about whether there are differences in disease severity associated with different determinants of socioeconomic position. Despite national guidance in the UK [6], referral pathways to paediatric rheumatology can be unclear and variable, and depend on multiple factors [7–9]. Longer disease duration at the time of commencing treatment may mean a ‘window of opportunity’ is missed, with consequences for long-term quality of life [10].

It is broadly recognized that health-related quality of life (HRQoL), including both physical and psychological aspects, is impacted by a diagnosis of a chronic condition [11], including amongst children and young people with JIA [12, 13]. In addition, socioeconomic position is associated with HRQoL amongst those with a chronic condition [14]. However, detailed understanding of HRQoL for children and young people with JIA by ethnic group and socioeconomic position remains limited.

Furthermore, given the apparent differences in incidence and prevalence of JIA by ethnic group, which may be associated with diagnostic delays, and the recognized variability in referral and diagnosis pathways in the UK, it is important to investigate the interrelationships between these factors—ethnicity, diagnostic delay and quality of life.

This study aimed to investigate the referral and initial treatment patterns, change in HRQoL following first rheumatology appointment, and clinical disease activity outcomes after 12 months, for children and young people with JIA in the UK by ethnic group and socioeconomic position.

## Patients and methods

### Study design

This analysis used data collected as part of the Childhood Arthritis Prospective Study (CAPS), an inception cohort for children with JIA between 2001 and 2019 described elsewhere [15]. In brief, children and young people aged <16 years presenting to one of seven paediatric and adolescent rheumatology referral centres across the UK with a new diagnosis of JIA were eligible to participate. The study was approved by the UK Northwest Multicentre Research Ethics Committee (MREC 02/8/104). Written informed consent from parents/guardians of all patients was obtained and all able children provided informed assent. Consent was obtained according to the Declaration of Helsinki.

### Data collection

Data were collected at first paediatric rheumatology appointment (baseline), after 6 months and annually for the first 5 years following presentation via medical case note review, patient questionnaires and interviews. At presentation, data collected included patient demographics, self-declared ethnicity, self-reported symptom duration, referral details (including dates and referral source), current disease activity measured using the JIA core outcome variables [16], and HRQoL via the Child Health Questionnaire (CHQ-50) [17]. In addition, whether patients had been diagnosed with another chronic health condition was recorded (including psoriasis, asthma, diabetes, uveitis, eczema or any other chronic condition). The physician assigned the ILAR subtype where appropriate [1]. Additional information collected at each follow-up include disease activity and CHQ-50.

### Ethnicity

Self-declared ethnicity was collected at recruitment and grouped according to the five first-tier ethnic group categories—White, Mixed, Asian, Black and Other–according to the Office for National Statistics England ethnic group classification [18].

### Indices of deprivation

The use of Indices of Multiple Deprivation (IMD) in CAPS has been previously described [19]. To summarize, patients were assigned to a nationwide deprivation rank using the most recent IMD [20], which is a composite indicator of relative deprivation across multiple domains, based on home address postcode at the time of JIA diagnosis. IMD was grouped into a dichotomous variable—patients living in the most deprived quintile of addresses compared with all other patients—to identify whether the most deprived communities have different outcomes compared with the rest of the population. The calculation of IMD scores differs between nations in the UK, therefore quintiles were determined separately for England (based on 2019 scores), Scotland (based on 2012 scores) and Wales (based on 2014 scores), then combined into an overall IMD quintile score; no patients were from Northern Ireland.

### Outcomes

The outcomes of this analysis investigated the referral pathway for children and young people with JIA, their HRQoL, and clinical outcomes, specifically looking at:

- Duration from symptom onset to initial referral, and from initial referral to first appointment in paediatric rheumatology.
- Association between symptom duration, initial disease presentation, referral source and presence of other chronic conditions with ethnic and IMD groups.
- Change in HRQoL following first appointment using the CHQ-50. The CHQ-50 is a validated parent/patient questionnaire comprising 50 questions, which gives scores for each of 14 physical and psychosocial domains (0–100, where higher scores indicate better health) and aggregates to two overall scores—physical health and psychological health.
- Clinical disease activity outcomes after 12 months. Clinical disease activity was assessed using the Juvenile Arthritis Disease Activity Score [21], based on 71-joint count (JADAS-71), and minimal disease activity (MDA) was calculated 12 months following first appointment [22].

### Patient inclusion and covariables

This analysis included newly diagnosed (treatment naïve) JIA patients with a confirmed physician’s diagnosis of JIA for whom ethnicity was recorded, registered between August 2001 and July 2019.

As this is an inception cohort, the final ILAR subtype is not always determined at first paediatric rheumatology appointment (i.e. initial JIA diagnosis), therefore the initial disease presentation (systemic, oligoarticular or polyarticular) is also described.

The presence of one or more chronic health conditions was coded as a single dichotomous variable. The rationale for this was to explore whether the presence of another chronic health condition influenced referral pathways or diagnosis delays.

### Analysis

Demographics and disease characteristics at recruitment were summarized for the total cohort and stratified by ethnic group and IMD group. Comparisons between groups were conducted using χ^2^ tests for categorical variables and Kruskal–Wallis tests for continuous variables.

Duration from symptom onset to referral, and referral to first appointment in paediatric rheumatology, were compared by initial disease presentation and categorized by ethnic group and IMD group using Kruskal–Wallis tests.

The association between duration, initial disease presentation, referral source and presence of other chronic conditions, and ethnic and IMD groups was assessed using Cox proportional hazards models, adjusted for age, gender, initial disease presentation, referral source, previous diagnosis of a chronic condition and, for time to first appointment, the time between onset and referral.

Mean CHQ-50 scores at baseline and after 12 months, and mean score changes between those time points were compared between ethnic groups and IMD categories using linear regression. Overall CHQ physical and CHQ psychosocial scores were also compared over 5 years.

The mean JADAS-71 at baseline and after 12 months, and mean change in JADAS-71 between those time points were compared between ethnic groups and IMD groups assessed using linear regression.

Multiple imputation using chained equations (72 datasets) was used to account for missing disease activity and covariate data. Complete variables included in the imputation model included age, gender, ethnic group, IMD and initial disease presentation category. Imputed disease activity variables included the core outcome variables that contribute to JADAS-71 (at baseline and 12 months) and CHQ scores (at baseline, 12, 36 and 60 months). Change in JADAS-71 and CHQ aggregate scores were calculated using imputed variables.

Analysis was performed using Stata Version 14.0.

## Results

### Baseline characteristics

In total 1275 children and young people with JIA met the inclusion criteria and were included in this analysis, of which 65% were female, 91% were in the White ethnic group (5% Asian, 1% Black and 3% Mixed ethnicity) and 27% were in the most deprived IMD quintile (Table 1). Median age at first presentation to paediatric rheumatology was 8 years [interquartile range (IQR) 4–12]. The most frequent ILAR category for all ethnic groups was persistent oligoarthritis (44% of all patients, range 36–50% by ethnic group). The most common initial disease presentation was oligoarticular (65%). Overall, 31% of patients reported having a diagnosis of another chronic condition at the time of their first appointment in paediatric rheumatology (the most frequent of which was asthma, diagnosed in 9% of patients), and 3% had symptoms of psoriasis. Missingness is presented in Supplementary Table S1.

**Table 1:** Characteristics of the 1275 children and young people within the cohort, at the time of their first appointment in paediatric rheumatology.

Characteristic		Whole cohort	Ethnic group					IMD group		
			White	Asian	Black	Mixed	*P*-value	Most deprived quintile	All others	***P*-value**
*N* (row %)		1275	1154 (91)	61 (5)	16 (1)	44 (3)		317 (27)	856 (73)	
Gender, *n* (%)	Male	426 (35)	385 (35)	18 (32)	7 (47)	16 (36)	0.745	110 (35)	316 (35)	0.875
	Female	795 (65)	720 (65)	39 (68)	8 (53)	8 (53)		202 (65)	593 (65)	
Age at first appointment with paediatric rheumatologist, median (IQR)		8 (4, 12)	7 (3, 12)	8 (4, 12)	10 (7, 12)	9 (4, 13)	0.380	9 (5, 12)	7 (3, 12)	**0.001**
IMD quintile, *n* (%)	Q1—Most deprived	317 (27)	272 (26)	22 (42)	6 (40)	17 (40)	**0.012**			
	Q2	248 (21)	222 (21)	14 (26)	5 (33)	7 (16)				
	Q3	199 (17)	181 (17)	8 (15)	2 (13)	8 (19)				
	Q4	183 (16)	173 (16)	5 (9)	0 (0)	5 (12)				
	Q5—Least deprived	226 (19)	214 (20)	4 (8)	2 (13)	6 (14)				
ILAR category, *n* (%)	Persistent oligoarticular	556 (44)	506 (44)	22 (36)	8 (50)	20 (45)	0.100	152 (48)	404 (42)	0.143
	Oligoarticular extended	64 (5)	57 (5)	4 (7)	0 (0)	3 (7)		10 (3)	56 (6)	
	Systemic	86 (7)	77 (7)	4 (7)	3 (19)	2 (5)		17 (5)	69 (7)	
	Polyarticular RF–	228 (18)	211 (18)	8 (13)	1 (6)	8 (18)		54 (17)	174 (18)	
	Polyarticular RF+	38 (3)	30 (3)	5 (8)	2 (13)	1 (2)		9 (3)	29 (3)	
	Psoriatic	95 (7)	90 (8)	5 (8)	0 (0)	0 (0)		21 (7)	74 (8)	
	Enthesitis-related	61 (5)	56 (5)	3 (5)	1 (6)	1 (3)		10 (3)	51 (5)	
	Undifferentiated	147 (12)	127 (11)	10 (16)	1 (6)	9 (20)		44 (14)	103 (11)	
Disease group at initial presentation, *n* (%)	Oligoarticular	823 (65)	745 (65)	39 (66)	9 (56)	30 (68)	0.657	207 (66)	616 (65)	0.524
	Polyarticular	362 (28)	330 (29)	16 (27)	4 (25)	12 (27)		92 (29)	270 (28)	
	Systemic	86 (7)	77 (7)	4 (7)	3 (19)	2 (5)		17 (5)	69 (7)	
History of another chronic condition, *n* (%)		390 (31)	350 (31)	20 (33)	3 (19)	17 (39)	0.462	97 (31)	293 (31)	0.996

Statistical tests: χ^2^ for categorical variables; Kruskal–Wallis test for continuous variables. IQR, interquartile range; IMD, Index of Multiple Deprivation. *P*-values in bold indicate statistical significance (*P*<.05).

### Duration from symptom onset to first appointment

The median time between symptom onset and first rheumatology appointment was 18 weeks (IQR 9–36), and varied somewhat by ethnic group (range 16–29 weeks; not significant) although not by IMD (Table 2). Median time from symptom onset to referral was 13 weeks (IQR 5–29), and from referral to first rheumatology appointment was 4 weeks (IQR 1–8).

**Table 2:** Referral and early treatment patterns, by ethnic group and socioeconomic position.

Characteristic	Whole cohort	Ethnic group					IMD group		
		White	Asian	Black	Mixed	*P*-value	Most deprived quintile	All others	*P*-value
N	1275	1154	61	16	44		317	958	
Time (weeks) from symptom onset to first paediatric rheumatology appointment, median (IQR)									
All cases	18 (9, 36)	18 (9, 35)	21 (12, 50)	29 (14, 46)	16 (9, 40)	0.307	17 (8, 34)	18 (9, 36)	0.288
Oligoarticular presentation	19 (9, 38)	18 (9, 37)	30 (13, 56)	38 (20, 46)	19 (9, 44)	0.142	18 (8, 38)	19 (9, 38)	0.303
Polyarticular presentation	20 (11, 37)	20 (11, 38)	18 (6, 33)	30 (23, 98)	11 (10, 24)	0.349	19 (11, 30)	21 (11, 40)	0.299
Systemic presentation	6 (3, 13)	6 (3, 13)	5 (1, 21)	8 (3, 14)	3 (1, 4)	0.634	5 (2, 14)	6 (3, 13)	0.558
Time (weeks) from symptom onset to referral to paediatric rheumatology, median (IQR)									
All cases	13 (5, 29)	13 (5, 29)	19 (6, 47)	20 (16, 94)	10 (5, 27)	0.111	12 (5, 28)	13 (6, 30)	0.417
Oligoarticular presentation	13 (5, 30)	12 (5, 29)	24 (9, 56)	33 (17, 94)	11 (5, 34)	**0.028**	12 (4, 32)	13 (6, 30)	0.316
Polyarticular presentation	15 (8, 29)	15 (8, 29)	13 (3, 24)	20 (17, 84)	10 (9, 11)	0.380	15 (9, 28)	15 (8, 33)	0.719
Systemic presentation	5 (2, 13)	5 (2, 14)	3 (1, 20)	3 (3, 3)	2 (1, 4)	0.455	4 (1, 11)	5 (2, 18)	0.450
Time (weeks) from referral to first paediatric rheumatology appointment, median (IQR)									
All cases	4 (1, 8)	4 (1, 8)	7 (3, 9)	5 (3, 11)	4 (1, 8)	**0.034**	4 (1, 8)	4 (2, 8)	0.137
Oligoarticular presentation	5 (2, 8)	4 (2, 8)	7 (3, 11)	5 (3, 11)	5 (2, 8)	0.087	5 (2, 9)	4 (2, 8)	0.648
Polyarticular presentation	4 (1, 8)	4 (1, 7)	7 (4, 9)	9 (5, 16)	2 (1, 12)	0.161	2 (0, 5)	4 (2, 8)	**< 0.001**
Systemic presentation	0 (0, 2)	0 (0, 2)	0 (0, 1)	2 (0, 3)	0 (0, 1)	0.861	0 (0, 1)	0 (0, 2)	0.655
Referral source, %									
General practitioner	17	17	16	13	11	0.970	20	16	**0.005**
Emergency department	7	7	7	0	5		10	6	
Orthopaedics	25	24	26	25	25		27	24	
Paediatrician	42	42	39	44	48		35	45	
Other	9	10	12	18	11		8	9	
First definitive treatment, *n* (%)									
IA joint injection	608 (56)	564 (57)	19 (41)	6 (46)	19 (49)	0.509	145 (54)	463 (57)	0.887
DMARD	299 (28)	262 (27)	19 (41)	4 (31)	14 (36)		77 (29)	222 (27)	
Biologic	6 (1)	5 (1)	0 (0)	0 (0)	1 (3)		2 (1)	4 0	
Systemic steroids	165 (15)	149 (15)	8 (18)	3 (23)	5 (13)		43 (16)	122 (15)	
All definitive treatments in first year, *n* (%)									
IA joint injection	900 (71)	826 (72)	33 (54)	9 (56)	32 (73)	**0.017**	212 (67)	688 (72)	0.094
DMARD	629 (49)	571 (49)	29 (48)	8 (50)	21 (48)	0.987	152 (48)	477 (50)	0.570
Biologic	142 (11)	131 (11)	6 (10)	0 (0)	5 (11)	0.539	27 (9)	115 (12)	0.087
Systemic steroids	403 (32)	370 (32)	17 (28)	5 (31)	11 (25)	0.707	107 (34)	296 (31)	0.343
Time (days) from first rheumatology appointment to first definitive treatment, median (IQR)									
Overall (any treatment)	13 (0, 38)	12 (0, 36)	13 (0, 50)	29 (3, 42)	18 (1, 52)	0.432	8 (0, 29)	15 (0, 40)	0.111
IA joint injection	19 (8, 46)	19 (8, 44)	19 (8. 124)	38 (29, 42)	26 (8, 55)	0.494	15 (5, 34)	21 (9, 47)	**0.022**
DMARD	0 (0, 33)	0 (0, 26)	5 (0, 40)	21 (0, 107)	29 (0, 49)	0.363	1 (0, 28)	0 (0, 35)	0.764
Biologic	18 (13, 29)	16 (13, 20)	*	*	*	0.143	23 (16, 29)	17 (7, 134)	0.643
Steroids	4 (0, 11)	4 (0, 10)	10 (2, 14)	3 (0, 6)	9 (0, 14)	0.569	5 (0, 11)	4 (0, 11)	1.000
Time (days) from first rheumatology appointment to first definitive treatment by initial disease presentation, median (IQR)									
Non-systemic presentation									
IA joint injection	19 (8, 44)	19 (8, 44)	19 (8, 124)	38 (29, 42)	26 (8, 55)	0.486	15 (5, 34)	21 (9, 47)	**0.022**
DMARD	0 (0, 35)	0 (0, 26)	5 (0, 40)	21 (0. 42)	29 (0, 49)	0.340	2 (0, 28)	0 (0, 36)	0.486
Biologic	*	*	*	*	*	0.179	*	*	0.655
Steroids	4 (0, 9)	4 (0, 13)	1 (7, 134)	*	*	0.698	2 (0, 7)	4 (0, 14)	0.296

Statistical tests: χ^2^ for categorical variables; Kruskal–Wallis test for continuous variables. DMARDs are those such as MTX. All definitive treatments in first year—*n* (%) of cohort who received this treatment within the first 12 months of their diagnosis with JIA; IQR, interquartile range; IMD, Index of Multiple Deprivation. * indicates fewer than three cases, so the data are suppressed due to median (IQR) being unrepresentative of the data. *P*-values in bold indicate statistical significance (*P*<.05).

**Figure 1:**
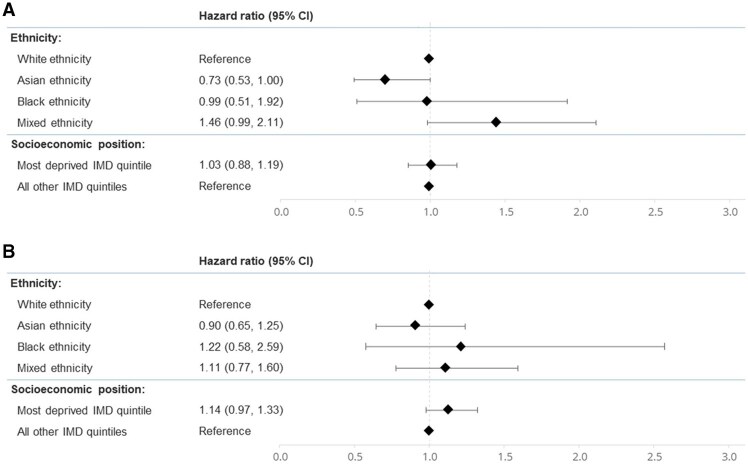
(A) Adjusted hazard ratios for time to referral to rheumatology from symptom onset and (B) adjusted hazard ratios for time to first paediatric rheumatology appointment following referral, in *N* = 1275 children and young people with JIA within this cohort

There was no difference in adjusted hazard ratios for duration between symptom onset and referral to paediatric rheumatology (Fig. 1A), nor for duration from referral to paediatric rheumatology to first appointment (Fig. 1B), by ethnic group or IMD group. Older age was associated with a longer time between symptom onset and referral, and age was a factor in referral to first rheumatology appointment (hazard ratio 0.93 per year of age, IQR 0.92–0.95), whilst longer symptom duration was associated with a longer time between referral and first rheumatology appointment. In contrast, those with systemic presentation of JIA tended to have a shorter time between symptom onset and referral, and from referral to first rheumatology appointment compared with those with oligoarticular presentation. History of another chronic condition was not associated with a longer wait time to referral, nor to first rheumatology appointment (Supplementary Table S2).

### Referral and initial treatment parameters

The highest proportion of patients were referred from a paediatrician (42%), followed by orthopaedics (25%); these proportions did not differ by ethnic group (Table 2), however a slightly lower proportion of those from the most deprived IMD quintile were referred by paediatrician (35% *vs* 45%).

Overall, 56% of patients’ first definitive treatment for their JIA was an IA joint injection, with 28% starting DMARDS such as MTX. This was similar between ethnic groups and IMD groups. During their first year following JIA diagnosis, 71% of patients had an IA joint injection; this ranged from 54% of Asian patients to 73% of patients with Mixed ethnicity. In addition, 49% of patients commenced a DMARD and 32% required systemic steroids within the first year.

The median interval between first paediatric rheumatology appointment and first treatment initiation was 13 days (IQR 0–38), which varied by treatment type (median time to start a DMARD was 0 days; median time to have a joint injection was 19 days).

### Health-related quality of life

Children and young people showed improvements between first paediatric rheumatology appointment and the 12-month assessment across most CHQ domains (Supplementary Table S3), although these remain below the published reference norms [17]. Whilst children and young people from White ethnic groups showed improvements in most domains (with the exception of general health, behaviour and family cohesion), changes for those from Asian, Black and Mixed ethnic groups were not significant for any CHQ domains, with the exception of ‘change’ (assessed as a single question comparing their child’s health to 1 year prior). There were no significant differences between ethnic groups or IMD groups in scores at baseline or 12 months, nor in change over time. However, the overall pattern of change over time appears to differ between ethnic groups, with mental health worsening for children of Asian ethnic group, and general health worsening for all ethnic groups except Mixed.

**Figure 2:**
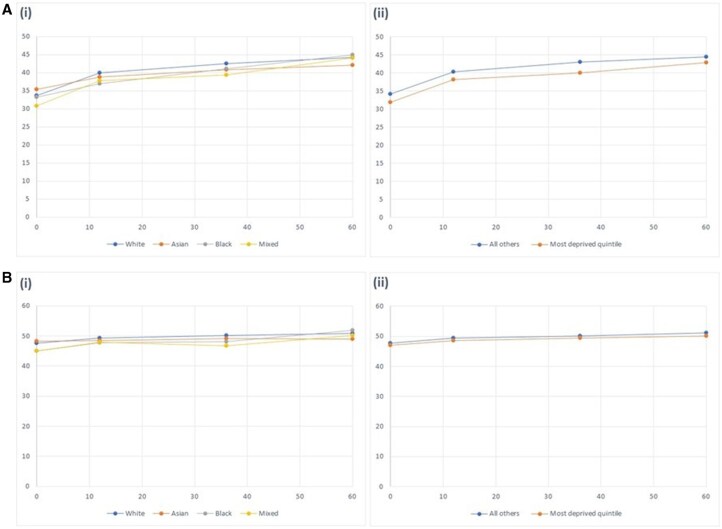
Measures of physical and psychosocial health over 5 years after diagnosis with JIA by ethnic group and socioeconomic position. (A) Line chart showing Child Health Questionnaire (CHQ) physical summary score by (i) ethnicity and (ii) Index of Multiple Deprivation (IMD) from baseline to 5 years. (B) Line chart showing CHQ psychosocial summary score by (i) ethnicity and (ii) IMD from baseline to 5 years

Overall scores for CHQ physical health were similar between ethnic groups and IMD categories, and exhibited slight increases at 1 year, but minimal change thereafter up to 5 years (Fig. 2A). Overall scores for psychosocial health were similar between ethnic groups and IMD groups and showed no increase over time (Fig. 2B).

### Clinical disease activity outcomes

Overall, patients exhibited improvements in measures of disease activity at 12 months (Table 3). Mean change in JADAS-71 at 12 months (95% CI) was broadly similar for all ethnic groups: –5.5 units (–6.4, –4.6) for patients of White ethnicity, –4.2 (–7.4, –0.9) for patients of Asian ethnicity, –1.5 (–8.5, 5.4) for patients of Black ethnicity and –3.5 (–7.8, 0.9) for patients of Mixed ethnicity. The proportion of children and young people who achieved MDA at 12 months (55%) was similar between ethnic groups and IMD groups.

**Table 3:** Disease activity measures at baseline and 12 months, by ethnic group and socioeconomic position.

Disease activity measure, mean (95% CI)	Whole cohort	Ethnic group					IMD group		
		White	Asian	Black	Mixed	*P*-value	Most deprived quintile	All others	*P*-value
*N*	1275	1154	61	16	44		317	958	
JADAS-71									
Baseline	17.4 (16.6, 18.1)	17.5 (16.7, 18.2)	16.4 (13.4, 19.3)	13.3 (8.0, 18.6)	17.1 (13.3, 21.0)	0.440	17.9 (16.5, 19.4)	17.2 (16.4, 18.0)	0.291
12 months	12.0 (11.6, 12.5)	12.0 (11.5, 12.5)	12.2 (10.1,14.3)	11.8 (7.3, 16.3)	13.7 (10.7, 16.6)	0.277	12.8 (11.9,13.7)	11.8 (11.2, 12.3)	0.061
Change	–5.3 (–6.2, –4.5)	–5.5 (–6.4, –4.6)	–4.2 (–7.4, –0.9)	–1.5 (–8.5, 5.4)	–3.5 (–7.8, 0.9)	0.188	–5.1 (–6.8, –3.5)	–5.4 (–6.3, –4.5)	0.880
Minimal disease activity^a^									
*N*	1214	1098	58	15	43		307	907	
12 months	55%	55%	50%	52%	59%	0.859	52%	56%	0.320

a Statistical tests: linear regression. Excludes patients with enthesitis-related arthritis. JADAS-71, 71-joint count Juvenile Arthritis Disease Activity Score; IMD, Index of Multiple Deprivation.

## Discussion

This is the first analysis using an inception cohort to investigate both physical and psychosocial outcomes from the initial diagnosis of JIA. We found no overall association between these scores and either ethnic group or socioeconomic position, although ethnic differences in children with JIA in the UK were reflected in variation across the deprivation index. In addition, it is the first analysis investigating time from symptom onset to referral, and from referral to paediatric rheumatology appointment, for children and young people with JIA by ethnic group and socioeconomic position. Overall, there was no evidence that duration from symptom onset to referral or from referral to appointment, or changes in disease activity, were associated with either ethnic group or socioeconomic position.

When looking at time between symptom onset and referral, and referral to first appointment, whilst ethnicity group and IMD were not associated, age and disease duration were, and this was not influenced by JIA phenotype. It was considered that patients with a diagnosis of another chronic health condition may be seen by health professionals more regularly as part of their routine care. However, this did not appear to reduce the duration between symptom onset referral and first rheumatology appointment.

Children and young people with systemic presentation of JIA tended to have a shorter duration (compared with oligoarticular presentation) both between symptom onset and referral, and between referral and first appointment, a trend previously reported [15, 23]; this analysis has found that this did not differ between ethnic groups or socioeconomic position.

In this analysis, the referral source represents the health professional that made the referral to paediatric rheumatology. This does not take into account other health professionals or services that may have been consulted prior to that referral. Nonetheless, this analysis highlights the complex and multifaceted referral pathway for children and young people with JIA into the paediatric rheumatology speciality, confirming the findings of others [8, 9]. Whilst those with the most severe presentations may be expected to attend an emergency department and be referred more quickly, this study identified that a referral via an emergency department tended to be instigated sooner following symptom onset than from a general practitioner (GP) and result in an earlier appointment, an association which persisted after adjusting for the three main disease presentation types. This finding (previously reported for children and young people overall [23]) also persisted after adjustment by ethnic group and socioeconomic position. Whilst this may reflect that a child presenting at an emergency department may be seen by specialist health services (such as rheumatology) within a hospital setting that are not available within a GP practice, there were no differences between ethnic groups or socioeconomic position identified.

The choice of first definitive treatment is typically made by the consulting physician, taking disease course and severity, national guidelines and local practice into account. Whilst current national treatment guidelines [6] do not include newer treatment options at disease onset, the initial therapies offered to patients remained unchanged and, reassuringly, there was no difference in choice of first treatment by ethnic group or socioeconomic position. In contrast, a study in the USA reported that children of Black ethnicity were more likely to be prescribed TNF inhibitor in the first year following diagnosis compared with patients of other ethnicities, although this apparent association disappeared once disease severity was accounted for [24].

The duration from first rheumatology appointment to first treatment initiation varied widely for the same treatment and same disease course. Whilst over half of those with non-systemic presentation started DMARD treatment on the day of their first rheumatology appointment, 25% had to wait more than 5 weeks before commencing treatment. As reported by national quality improvement audits [8, 9] these differences may have an impact on later JIA outcomes and quality of care; however, in this study we have not identified any associations between ethnicity or deprivation that relate to this variability.

In this cohort, the overall measures of parent/patient reported physical health were substantially lower than in the general population throughout the first 5 years following diagnosis, indicating poorer health than the published reference ‘norms’ [17] (baseline 34, 12 months 40; compared with a reference norm of 53). Psychosocial health also remained lower than published reference values but to a lesser extent, indicating the substantial impact that JIA has on all aspects of a patient’s life. Initial improvements in CHQ appear to be greatest in the physical functioning, physical and bodily pain/discomfort domains, which may reflect aspects most immediately impacted by early treatment. Additionally, the parental emotional health domain showed improvements at twelve months following first rheumatology appointment, potentially due to receiving a diagnosis, commencing treatment and seeing improvements in physical health of their child. However, it is noticeable that neither physical nor psychosocial health appeared to continue to improve substantially for patients beyond the first year, but remained largely stable, a pattern seen both in other chronic conditions of childhood and adult-onset RA [25, 26]. In our study, this pattern was similar between ethnic groups and between IMD groups, which contrasts with other studies which reported higher CHQ scores for patients of White ethnicity compared with other ethnic groups [27].

This study identified improvements in JADAS-71 for all ethnic groups and socioeconomic groups following diagnosis with JIA, with measures at both first presentation and after 12 months, as well as overall improvement in disease activity measures over time, broadly similar between patients. However, despite this the overall patient/parent reported physical and psychosocial health scores remained low. Studies have shown [13] that children with JIA experience long-term substantial mental health impacts, and these may contribute to the low scores in this study despite improvement in disease activity. Similarly, improvement in HRQoL has previously been reported as being slower than improvement in disease activity [12].

In this study improvements in disease activity using JADAS-71 were similar between ethnic groups, which has previously been reported for children using JADAS-10 (the different versions of JADAS have been validated together [21]) as part of a treat-to-target approach [28]. In a study in Canada [29], South Asian ethnicity was associated with shorter time to an first appointment in paediatric rheumatology, in contrast to our findings where ethnicity was not associated with duration from symptom onset to referral nor to first appointment.

This analysis was completed in a large national inception cohort of children and young people with JIA, involving wide data capture and strong statistical methodology. However, limitations of this study include the relatively small sample size within the Asian, Black and Mixed ethnic groups, which lead to increased variability and decreased statistical power. In addition, the proportion of children and young people of White ethnicity (91%) was higher than has been reported in the general population (73%) [30]. Whilst patients were recruited from specialist rheumatology centres, it is possible that they may not be entirely representative of the population of children and young people with JIA as a whole. It is not possible to account for travel time or distance to appointments, which may have influenced some of the findings. This limits our ability to make broader conclusions. Whilst the use of multiple imputation seeks to minimize the impact of missing data on selection bias, some variables had a high level of missing data which may affect the precision of estimates. Furthermore, the time of symptom onset was self-reported by the patient or parent, and therefore may not accurately represent the onset of disease activity. Whilst patients were recruited to the study at the point of first rheumatology appointment and therefore naïve to DMARD treatment, some may have had prior NSAID treatment which is readily available without a prescription in the UK, or via their GP. Information about prior NSAID use is not captured within the dataset. The degree to which NSAID use may have differed between groups and any impact of disease activity is not known.

Importantly and reassuringly, neither ethnicity nor socioeconomic position appear to be associated with referral and initial treatment patterns, patient-reported health-related quality of life measures, or improvements in disease activity. Overall, the findings suggest no detectable differences between ethnic groups within the context of available sample size. It appears that the majority of children and young people with JIA in this inception cohort experience improvements in measures of disease activity following first rheumatology appointment. However, despite this, overall patient/parent-reported measures show poor outcomes for both physical and psychosocial scores. Further research is warranted to understand the low parent/patient reported physical and psychosocial scores for children with JIA with apparent low disease activity, in order to target interventions to improve overall quality of life in these individuals.

## Supplementary Material

keag332_Supplementary_Data

## Data Availability

The data underlying this article cannot be shared publicly to maintain the privacy of the individuals who participated in the study. Information regarding applying for access to CAPS data can be found online, available at www.caps-jia.org.uk/clinicians-and-researchers/.

## Patient involvement

As part of a wider project, in preparing for this analysis, the authors discussed the proposed project through focus groups and interviews with parents of children and young people with JIA, from a diverse range of ethnic backgrounds. This analysis reflects the priorities identified by a diverse population, including whether duration to first appointment in paediatric rheumatology, and psychosocial outcomes, are associated with ethnicity and broader social determinants of health.
