# Supplementary material for: Referral, treatment patterns and change in quality of life in the first 12 months in children and young people with juvenile idiopathic arthritis: an analysis of the association with ethnicity and socioeconomic position using data from a cohort study
Source: Rheumatology (Oxford). 2026 Jun 25;65(7):keag332. doi: 10.1093/rheumatology/keag332 (PMC13387603; doi:10.1093/rheumatology/keag332)
Supplement: keag332_Supplementary_Data [file keag332_supplementary_data.docx]

**Supplementary Table S1**. Proportion of missing data from demographic and core outcome variables of the 1275 children and young included in the analyses.

| **Variable** | **Ethnic Group** | | | |  | **IMD Group** | | |
| --- | --- | --- | --- | --- | --- | --- | --- | --- |
|  | **White** | **Asian** | **Black** | **Mixed** |  | **Most deprived quintile** | **All others** |  |
| Number of patients | 1154 | 61 | 16 | 44 |  | 317 | 958 |  |
| Gender | 4% | 7% | 6% | 0% |  | 2% | 5% |  |
| Age at baseline | 4% | 7% | 6% | 0% |  | 2% | 5% |  |
| Index of Multiple Deprivation (IMD) | 0% | 0% | 0% | 0% |  | 0% | 0% |  |
| ILAR category | 0% | 0% | 0% | 0% |  | 0% | 0% |  |
| Disease duration | 26% | 30% | 38% | 27% |  | 20% | 29% |  |
| Referral source | 0% | 0% | 0% | 0% |  | 0% | 0% |  |
| Child Health Questionnaire (CHQ), baseline | 58% | 74% | 88% | 64% |  | 58% | 60% |  |
| Child Health Questionnaire (CHQ),12 months | 58% | 74% | 75% | 59% |  | 63% | 58% |  |

**Supplementary Table S2**. Adjusted hazard ratios for duration between (A) symptom onset and referral to paediatric rheumatology, and (B) from referral to paediatric rheumatology and first appointment, in children and young people with JIA within this cohort.

| **Variable** | **Adjusted hazard ratio (95% CI)** | |
| --- | --- | --- |
|  | **(A) symptom onset and referral to paediatric rheumatology** | **(B) referral to paediatric rheumatology and first appointment** |
| **Ethnic group** |  |  |
| White | Reference | Reference |
| Asian | 0.73 (0.53, 1.00) | 0.90 (0.65, 1.25) |
| Black | 0.99 (0.51, 1.92) | 1.22 (0.58, 2.59) |
| Mixed | 1.46 (0.99, 2.11) | 1.11 (0.77, 1.60) |
| **Socioeconomic position** |  |  |
| Most deprived IMD group | 1.03 (0.88, 1.19) | 1.14 (0.97, 1.33) |
| All other IMD quintiles | Reference | Reference |
| **Age (years)** | 0.93 (0.92, 0.95) | 0.96 (0.94, 0.98) |
| **Gender** |  |  |
| Male | Reference | Reference |
| Female | 0.91 (0.79, 1.05) | 0.97 (0.84, 1.13) |
| **Initial disease presentation** |  |  |
| Oligoarticular presentation | Reference | Reference |
| Polyarticular presentation | 0.87 (0.75, 1.02) | 1.11 (0.95, 1.31) |
| Systemic presentation | 2.06 (1.59, 2.67) | 4.02 (2.95, 5.49) |
| **Referral source** |  |  |
| GP | Reference | Reference |
| Emergency department | 2.75 (2.04, 3.69) | 1.85 (1.35, 2.53) |
| Orthopaedics | 1.19 (0.97, 1.47) | 1.03 (0.83, 1.27) |
| Paediatrician | 1.15 (0.94, 1.40) | 1.24 (1.01, 1.53) |
| Other healthcare professional | 0.92 (0.69, 1.23) | 1.43 (1.07, 1.93) |
| **History of chronic condition** | 0.97 (0.84, 1.12) | 0.98 (0.85, 1.15) |
| **Duration from onset to referral**, weeks | n/a | 0.99 (0.99, 0.99) |

**Supplementary Table S3**. Descriptive summary of CHQ domains by ethnic group and IMD group at first paediatric rheumatology appointment and after 12 months.

| **CHQ domain, mean (95% CI)** | **Ethnic Group** | | | |  | **IMD Group** | | |  |
| --- | --- | --- | --- | --- | --- | --- | --- | --- | --- |
|  | **White** | **Asian** | **Black** | **Mixed** | **p-value** | | **Most deprived quintile** | **All others** | **p-value** |
| N | 1154 | 61 | 16 | 44 |  | | 317 | 856 |  |
|  |  |  |  |  |  | |  |  |  |
| **Global health** |  |  |  |  |  | |  |  |  |
| Baseline | 68.4 (66.1, 70.8) | 68.9 (59.9, 77.9) | 68.6 (51.1, 86.2) | 70.4 (59.4, 81.4) | 0.733 | | 68.9 (66.5, 71.3) | 68.9 (66.5, 71.3) | 0.493 |
| 12 months | 69.4 (67.3, 71.5) | 67.8 (58.6, 77.1) | 63.2 (45.2, 81.3) | 73.4 (63.2, 82.0) | 0.749 | | 67.0 (63.2, 70.9) | 70.1 (67.9, 71.6) | 0.153 |
| Change | 0.9 (-1.8, 3.7) | 1.1 (-13.6, 11.7) | 5.2 (-30.9, 20.4) | 3.0 (-9.7, 15.7) | 0.992 | | -0.4 (-5.7, 4.9) | 1.2 (-1.7, 4.1) | 0.602 |
|  |  |  |  |  |  | |  |  |  |
| **Physical functioning** |  |  |  |  |  | |  |  |  |
| Baseline | 63.9 (61.3, 66.6) | 65.8 (54.4, 77.1) | 63.9 (39.7, 88.0) | 59.2 (45.9, 72.4) | 0.629 | | 60.3 (55.3, 65.2) | 65.0 (62.2, 67.9) | 0.083 |
| 12 months | 75.2 (73.0, 77.6) | 72.8 (61.9, 83.8) | 74.2 (54.3, 94.1) | 71.0 (58.3, 83.7) | 0.447 | | 71.3 (67.1, 75.5) | 76.2 (73.6, 78.8) | **0.041** |
| Change | 11.3 (8.2, 14.5) | 7.2 (-8.2, 22.4) | 10.3 (-19.7, 40.4) | 11.8 (-4.5, 28.2) | 0.881 | | 11.0 (5.1, 17.0) | 11.2 (7.7, 14.7) | 0.966 |
|  |  |  |  |  |  | |  |  |  |
| **Role – Emotional / behavioural** |  |  |  |  |  | |  |  |  |
| Baseline | 76.7 (74.1, 79.3) | 78.0 (67.4, 88.4) | 74.8 (52.3, 97.3) | 70.4 (57.0, 83.8) | 0.434 | | 73.4 (68.6, 78.2) | 77.5 (74.7, 80.3) | 0.132 |
| 12 months | 82.6 (80.4, 84.8) | 78.8 (68.4, 89.2) | 79.5 (60.8, 98.3) | 80.9 (68.4, 93.3) | 0.580 | | 79.3 (75.1, 83.6) | 83.3 (80.9, 85.7) | 0.088 |
| Change | 5.9 (2.9, 8.9) | 0.9 (-13.4, 15.2) | 4.8 (-24.1, 33.6) | 10.5 (-6.4, 27.3) | 0.813 | | 5.9 (-0.1, 12.0) | 5.8 (2.5, 9.1) | 0.970 |
|  |  |  |  |  |  | |  |  |  |
| **Role – Physical** |  |  |  |  |  | |  |  |  |
| Baseline | 66.1 (63.1, 69.2) | 69.2 (57.3, 81.1) | 65.6 (39.8, 91.4) | 59.2 (44.2, 74.3) | 0.549 | | 62.7 (57.5, 68.0) | 67.1 (63.8, 70.5) | 0.146 |
| 12 months | 77.3 (74.9, 79.8) | 73.5 (61.7, 85.3) | 70.9 (49.1, 92.6) | 69.4 (54.8, 84.0) | 0.172 | | 73.9 (69.0, 78.8) | 77.8 (75.1, 80.4) | 0.151 |
| Change | 11.2 (7.7, 14.6) | 4.3 (-11.8, 20.3) | 5.2 (-27.1, 37.5) | 10.2 (-8.0, 28.3) | 0.622 | | 11.2 (4.5, 17.8) | 10.6 (6.7, 14.5) | 0.886 |
|  |  |  |  |  |  | |  |  |  |
| **Bodily pain / discomfort** |  |  |  |  |  | |  |  |  |
| Baseline | 48.1 (45.8, 50.3) | 53.6 (43.3, 64.0) | 50.0 (28.8, 71.1) | 44.7 (33.3, 56.2) | 0.928 | | 46.2 (42.1, 50.3) | 48.9 (46.4, 51.5) | 0.255 |
| 12 months | 60.6 (58.3, 62.9) | 58.7 (48.5, 69.0) | 54.7 (35.4, 74.1) | 58.5 (46.3, 70.7) | 0.545 | | 57.6 (53.4, 61.9) | 61.2 (58.7, 63.7) | 0.143 |
| Change | 12.5 (9.6, 15.4) | 5.1 (-8.1, 18.3) | 4.8 (-23.7, 33.2) | 13.8 (-1.9, 29.4) | 0.700 | | 11.4 (5.9, 16.9) | 12.3 (9.1, 15.5) | 0.781 |
|  |  |  |  |  |  | |  |  |  |
| **Behaviour** |  |  |  |  |  | |  |  |  |
| Baseline | 69.8 (67.9, 71.7) | 70.9 (64.2, 77.6) | 66.7(52.0, 81.4) | 67.2 (58.5, 76.0) | 0.582 | | 69.0 (66.0, 72.0) | 70.0 (67.9, 72.0) | 0.562 |
| 12 months | 70.7 (61.1, 76.1) | 68.6 (61.1, 76.1) | 68.5 (55.1, 81.8) | 70.0 (60.7, 78.4) | 0.651 | | 68.9 (66.0, 71.8) | 71.0 (69.1, 72.9) | 0.193 |
| Change | 0.9 (-1.2, 2.9) | -2.3 (-11.9, 7.3) | 1.8 (-17.4, 20.9) | 2.3 (-8.8, 13.5) | 0.953 | | -0.1 (-3.4, 3.8) | 1.1 (-1.2, 3.4) | 0.599 |
|  |  |  |  |  |  | |  |  |  |
| **Mental health** |  |  |  |  |  | |  |  |  |
| Baseline | 72.0 (70.5, 73.6) | 72.8 (66.1, 79.5) | 68.1 (53.1, 83.1) | 66.9 (58.3, 75.4) | 0.241 | | 70.7 (67.9, 73.5) | 72.2 (70.5, 74.0) | 0.319 |
| 12 months | 75.3 (73.7, 76.9) | 72.9 (66.5, 79.3) | 73.3 (60.1, 86.4) | 72.2 (64.5, 80.0) | 0.386 | | 73.7 (71.0, 76.5) | 75.5 (73.9, 77.1) | 0.257 |
| Change | 3.3 (1.4, 5.2) | 0.1 (-8.7, 8.8) | 5.1 (-13.5, 23.8) | 5.4 (-5.0, 15.8) | 0.844 | | 3.1 (-0.6, 6.7) | 3.3 (1.2, 5.4) | 0.920 |
|  |  |  |  |  |  | |  |  |  |
| **Self-esteem** |  |  |  |  |  | |  |  |  |
| Baseline | 69.9 (68.1, 71.8) | 71.6 (63.8, 79.4) | 61.2 (48.6, 79.7) | 65.3 (56.0, 74.6) | 0.344 | | 69.6 (66.5, 72.7) | 69.7 (67.8, 71.9) | 0.876 |
| 12 months | 73.7 (72.0, 75.5) | 76.7 (69.4, 83.9) | 69.2 (54.5, 83.9) | 68.2 (59.2, 77.2) | 0.347 | | 72.5 (69.3, 75.6) | 74.0 (72.2, 75.8) | 0.378 |
| Change | 3.8 (1.7, 5.9) | 5.0 (-4.9, 15.0) | 5.1(-16.7, 28.8) | 2.9 (-8.7, 14.5) | 0.992 | | 2.9 (-1.3, 7.0) | 4.1 (1.8, 6.5) | 0.612 |
|  |  |  |  |  |  | |  |  |  |
| **General health perceptions** |  |  |  |  |  | |  |  |  |
| Baseline | 60.9 (59.1, 62.7) | 60.8 (53.4, 68.2) | 58.2 (42.7, 73.7) | 62.2 (53.4, 70.8) | 0.904 | | 60.4 (57.3, 63.5) | 61.0 (59.1, 63.0) | 0.712 |
| 12 months | 58.8 (57.1, 60.5) | 59.1 (52.0 (66.2) | 56.9 (43.2, 70.6) | 62.2 (53.3, 71.2) | 0.589 | | 58.1 (55.2, 61.1) | 59.2 (57.3, 61.0) | 0.546 |
| Change | -2.1 (-4.2, 0.1) | -1.7 (-11.1, 7.7) | -1.3 (-21.9, 19.4) | 0.1 (-10.8, 11.0) | 0.699 | | -2.3 (-6.3, 1.8) | -1.9 (-4.2, 0.5) | 0.865 |
|  |  |  |  |  |  | |  |  |  |
| **Change in health** |  |  |  |  |  | |  |  |  |
| Baseline | 2.6 (2.5, 2.7) | 2.9 (2.5, 3.3) | 3.0 (2.1, 4.0) | 2.7 (2.2, 3.3) | 0.393 | | 2.7 (2.5, 2.9) | 2.7 (2.5, 2.8) | 0.747 |
| 12 months | 3.5 (3.4, 3.6) | 3.5 (3.1, 3.9) | 3.1 (2.3, 3.9) | 3.6 (3.1, 4.0) | 0.909 | | 3.3 (3.2, 3.5) | 3.5 (3.4, 3.6) | 0.070 |
| Change | 0.8 (0.7, 1.0) | 0.6 (0.1, 1.2) | 0.9 (-1.0, 1.2) | 0.8 (0.2, 1.5) | 0.516 | | 0.6 (0.4, 0.9) | 0.9 (0.7, 1.0) | 0.137 |
|  |  |  |  |  |  | |  |  |  |
| **Parental impact - emotional** |  |  |  |  |  | |  |  |  |
| Baseline | 60.9 (58.7, 63.1) | 65.5 (56.3 (74.6) | 59.3 (40.6, 78.0) | 59.1 (47.7, 70.6) | 0.963 | | 61.4 (57.4, 65.5) | 60.9 (58.5, 63.3) | 0.813 |
| 12 months | 68.9 (66.8, 71.1) | 68.9 (60.1, 77.6) | 67.5 (50.2, 87.7) | 69.5 (59.1, 79.8) | 0.983 | | 68.5 (65.9, 72.0) | 69.1(66.8, 71.4) | 0.779 |
| Change | 8.0 (5.4, 10.6) | 3.4 (-8.2, 15.0) | 8.2 (-15.8, 35.1) | 10.3 (-4.1, 24.8) | 0.958 | | 7.0 (2.1, 12.0) | 8.2 (5.2, 11.1) | 0.705 |
|  |  |  |  |  |  | |  |  |  |
| **Parental impact - time** |  |  |  |  |  | |  |  |  |
| Baseline | 77.6 (75.3, 79.8) | 77.2 (67.5, 86.9) | 74.3 (53.2, 95.3) | 74.3 (62.7, 86.0) | 0.575 | | 75.5 (71.7, 79.4) | 78.0 (75.6, 80.4) | 0.243 |
| 12 months | 82.7 (80.7, 84.7) | 79.6 (70.5, 88.8) | 79.1 (62.6, 95.6) | 79.5 (68.8, 90.3) | 0.408 | | 81.2 (77.7, 84.8) | 82.8 (80.6, 84.9) | 0.450 |
| Change | 5.1 (2.4, 7.9) | 2.4 (-10.8, 15.6) | 4.9 (-20.3, 30.1) | 5.2 (-8.9, 19.3) | 0.894 | | 5.7 (0.8, 10.7) | 4.8 (1.9, 7.6) | 0.736 |
|  |  |  |  |  |  | |  |  |  |
| **Family activities** |  |  |  |  |  | |  |  |  |
| Baseline | 70.7 (68.6, 72.8) | 72.2 (63.4, 81.0) | 69.2 (504, 87.9) | 67.3 (56.8, 77.9) | 0.627 | | 68.3 (64.5, 72.0) | 71.4 (69.2, 73.7) | 0.135 |
| 12 months | 77.2(75.2, 79.3) | 74.9 (66.3, 83.5) | 73.2 (58.1, 88.2) | 77.5 (67.4, 87.6) | 0.780 | | 75.9 (72.6, 79.2) | 77.5 (75.2, 72.0) | 0.415 |
| Change | 6.5 (4.1, 8.9) | 2.7 (-8.8, 14.2) | 4.0 (-18.8, 26.8) | 10.1 (-3.1, 23.3) | 0.842 | | 7.6 (2.9, 12.3) | 6.0 (3.3, 8.8) | 0.566 |
|  |  |  |  |  |  | |  |  |  |
| **Family cohesion** |  |  |  |  |  | |  |  |  |
| Baseline | 77.3 (75.4, 79.2) | 77.5 (70.3, 84.7) | 72.0 (55.3, 88.8) | 74.3 (65.4, 83.2) | 0.458 | | 77.1 (73.8, 80.3) | 77.2 (75.1, 79.3) | 0.939 |
| 12 months | 76.8 (74.9, 79.7) | 77.4 (69.8, 85.1) | 76.8 (62.0, 91.7) | 76.4 (67.4, 85.4) | 0.982 | | 76.3 (73.0, 79.5) | 77.0 (75.0, 79.0) | 0.698 |
| Change | -0.5 (-2.8, 1.7) | -0.1 (-10.3, 10.2) | 4.8 (-16.5, 26.1) | 2.1 (-9.5, 13.7) | 0.565 | | -0.8 (-4.9, 3.3) | -0.2 (-2.8, 2.4) | 0.814 |
|  |  |  |  |  |  | |  |  |  |
| **Summary physical score** |  |  |  |  |  | |  |  |  |
| Baseline | 33.7 (32.2, 35.2) | 35.5 (29.0, 41.9) | 33.4 (20.5, 46.4) | 31.2 (23.8, 38.6) | 0.701 | | 32.0 (29.3, 34.7) | 34.3 (32.7, 35.9) | 0.135 |
| 12 months | 40.0 (38.7, 41.4) | 38.9 (32.7, 45.1) | 37.4 (26.5, 48.3) | 37.7 (30.3, 45.2) | 0.424 | | 38.1 (35.6, 40.6) | 40.5 (39.0, 41.9) | 0.098 |
| Change | 6.3 (4.6, 8.1) | 3.4 (-4.9, 11.8) | 3.9 (-12.5, 20.4) | 6.5 (-2.6, 15.6) | 0.776 | | 6.1 (2.8, 9.4) | 6.2 (4.2, 8.2) | 0.971 |
|  |  |  |  |  |  | |  |  |  |
| **Summary psychosocial score** |  |  |  |  |  | |  |  |  |
| Baseline | 47.5 (46.6, 48.5) | 48.3 (44.5, 52.0) | 44.9 (36.4, 53.5) | 44.9 (40.1, 49.9) | 0.309 | | 47.0 (45.4, 48.6) | 47.6 (46.5, 48.7) | 0.551 |
| 12 months | 49.3 (48.4, 50.2) | 48.6 (44.8, 52.3) | 47.7 (40.2, 55.2) | 47.9 (43.2, 52.5) | 0.482 | | 48.5 (46.9, 50.0) | 49.4 (48.4, 50.4) | 0.287 |
| Change | 1.8 (0.7, 2.9) | 0.3 (-4.8, 5.4) | 2.8 (-8.1, 13.6) | 2.9 (-3.1, 8.9) | 0.831 | | 1.5 (-0.6, 3.5) | 1.8 (0.6, 3.1) | 0.758 |

Statistical tests: Linear regression.

CI – confidence interval. IMD – Index of Multiple Deprivation.
